# Biosensors for Brain Trauma and Dual Laser Doppler Flowmetry: Enoxaparin Simultaneously Reduces Stroke-Induced Dopamine and Blood Flow while Enhancing Serotonin and Blood Flow in Motor Neurons of Brain, *In Vivo*

**DOI:** 10.3390/s11010013

**Published:** 2010-12-24

**Authors:** Patricia A. Broderick, Edwin H. Kolodny

**Affiliations:** 1 Department Physiology & Pharmacology, Sophie Davis School of Biomedical Education, CCNY, New York, NY 10031, USA; 2 Departments Biology, Psychology, City University of New York Graduate School, New York, NY 10031, USA; 3 Department. Neurology, New York University Langone Medical Center, NYU Comprehensive Epilepsy Center, New York, NY 10031, USA

**Keywords:** enoxaparin, heparin, blood clots, acute ischemic stroke, motor neurons, dorsal striatum, dopamine, serotonin, homovanillic acid, L-Tryptophan, neuromolecular imaging, electrochemistry, *in vivo* microvoltammetry, cerebral blood flow, reperfusion, middle cerebral artery occlusion, edema, glycoprotein IIb/IIIa inhibitors, Factor Xa, Factor II, Factor Xa inhibitors, thrombosis, thrombospondin, monoclonal antibodies, platelets, anticoagulants, integrin, dual laser Doppler flowmetry, optic fiber, biochemical sensors, laser sensors

## Abstract

Neuromolecular Imaging (NMI) based on adsorptive electrochemistry, combined with Dual Laser Doppler Flowmetry (LDF) is presented herein to investigate the brain neurochemistry affected by enoxaparin (Lovenox^®^), an antiplatelet/antithrombotic medication for stroke victims. NMI with miniature biosensors enables neurotransmitter and neuropeptide (NT) imaging; each NT is imaged with a response time in milliseconds. A semiderivative electronic reduction circuit images several NT’s selectively and separately within a response time of minutes. Spatial resolution of NMI biosensors is in the range of nanomicrons and electrochemically-induced current ranges are in pico- and nano-amperes. Simultaneously with NMI, the LDF technology presented herein operates on line by illuminating the living brain, in this example, in dorso-striatal neuroanatomic substrates via a laser sensor with low power laser light containing optical fiber light guides. NMI biotechnology with BRODERICK PROBE^®^ biosensors has a distinct advantage over conventional electrochemical methodologies both in novelty of biosensor formulations and on-line imaging capabilities in the biosensor field. NMI with unique biocompatible biosensors precisely images NT in the body, blood and brain of animals and humans using characteristic experimentally derived half-wave potentials driven by oxidative electron transfer. Enoxaparin is a first line clinical treatment prescribed to halt the progression of acute ischemic stroke (AIS). In the present studies, BRODERICK PROBE^®^ laurate biosensors and LDF laser sensors are placed in dorsal striatum (DStr) dopaminergic motor neurons in basal ganglia of brain in living animals; basal ganglia influence movement disorders such as those correlated with AIS. The purpose of these studies is to understand what is happening in brain neurochemistry and cerebral blood perfusion after causal AIS by middle cerebral artery occlusion *in vivo* as well as to understand consequent enoxaparin and reperfusion effects actually while enoxaparin is inhibiting blood clots to alleviate AIS symptomatology. This research is directly correlated with the medical and clinical needs of stroke victims. The data are clinically relevant, not only to movement dysfunction but also to the depressive mood that stroke patients often endure. These are the first studies to image brain neurotransmitters while any stroke medications, such as anti-platelet/anti-thrombotic and/or anti-glycoprotein are working in organ systems to alleviate the debilitating consequences of brain trauma and stroke/brain attacks.

## 1. Introduction

**Figure 1.**
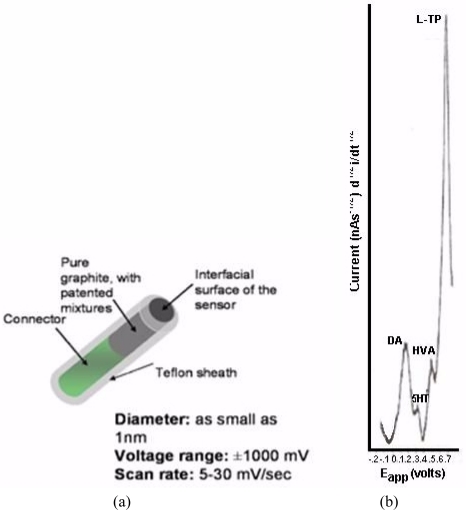
(**a**) BRODERICK PROBE^®^ (**b**) Typical baseline signatures for NMI recording of neurotransmitters (NT) in dorsal striatum (DStr); x axis is potential in millivolts, y axis is current as defined by the semiderivative reduction circuit, discovered in this laboratory (52). Symbols: DA is dopamine, 5-HT is serotonin, HVA is homovanillic acid and L-TP is L-Tryptophan.

NMI with BRODERICK PROBE^®^ biosensors selectively detect neurotransmitters, metabolites and precursors of neurotransmitters, excitatory and inhibitory neuropeptides (NT) *in vivo* in specific neuroanatomic substrates of brain, body and blood. In the present studies, dopaminergic motor neurons in basal ganglia of brain within DStr are selected because these dopaminergic neurons influence movement behaviors and disorders. The biogenic and indoleamines, dopamine (DA) and serotonin (5-HT), metabolites and precursors are separately imaged within the same recording with BRODERICK PROBE^®^ laurate biosensors at the same time as dual laser Doppler flowmetry monitored cerebral blood perfusion within the same dorso-striatal neuroanatomic substrate. NMI provides advantages over spectroscopic or chromatographic methods because NMI studies are performed in the complex living matrix *in vivo*. A schematic of the biosensor is shown in Figure 1a. The biosensor is comprised of fatty acids and lipids, normal constituents of human and animal brain [1,2]. The biosensor is used here to investigate the neuronal mechanism of action for stroke; in addition, the biosensor is used here to investigate enoxaparin, a first-line medication for stroke patients. Figure 1b is a typical baseline recording for NT’s studied in the present paper.

The basal ganglia is a complex neuroanatomic substrate targeted by stroke; it is comprised of paired subcortical masses or nuclei of gray matter that include the caudate putamen and the globus pallidum. The caudate and putamen are structurally distinct in the human and these structures are joined in lower mammals and are generally considered to function as a unit, called striatum. The terminology, striatum, is derived from the striped nature of this neuroanatomic substrate. Coursing through the striatum and closely related is the internal capsule which separates striatum from the lenticular nucleus. Also closely associated with the basal ganglia nuclei are small brain-stem nuclei, the substantia nigra, the ventral tegmentum and the subthalamus. The control of voluntary movement is executed by the interaction of the pyramidal, cerebellar, and extrapyramidal systems, which interconnect with each other as well as projecting to the cranial nerve nuclei.

**Figure 2.**
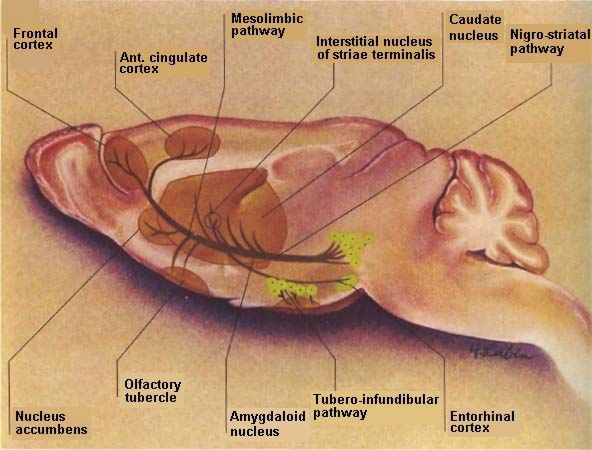
Depicted: Adapted diagram of DA pathways in basal ganglia in rat brain [3,4].

DA is a critical neurotransmitter modulator that projects from the substantia nigra *pars compacta* and *pars reticulata* to the striatum (A9) nigrostriatal pathway with analogues mesolimbic (A10) neurons eminating from the ventral tegmental area to what is commonly called, “the reward center”, the nucleus accumbens. It is interesting to note that the ventral tegmental “reward” center is also a target for stroke victims just as is the DStr, the movement entity as well as the cortical region, the cognitive performance entity. Thus, it is difficult for stroke patients to feel reward, to have normal movement, to speak about their feelings or to relate to another for cognitive communication (See Figure 2).

These small gray-matter basal nuclei, although they lie deep within the forebrain and hindbrain and yet, away from parts of the cortical area, have multi-faceted neuronal connections with the cortex. Electrophysiological studies in primates in addition to movement and cognitive studies in patients with dysfunctional movement, have shown that the basal nuclei operate to assist in movement to (a) determine force and velocity (b) prepare for movement (c) develop automaticity (d) promote sequential movement (e) inhibit unwanted movement (f) adapt to novel or reward movement and (g) motor learning and planning [5].

The indoleamine, serotonin (5-HT) is present within the DA pathways; immunocytoflourescent images of the co-presence of DA and 5-HT in ventral striatal DA pathways are published by this laboratory [6]. The dorsal (A9) and ventral (A10) DA pathways have been a major focus of study in this laboratory. Precise distinctions between and within the dorsal and ventral striatal substrates, as delineated by a number of different formulations of the BRODERICK PROBE^®^ microelectrode/biosensor, are published [7,8].

What is stroke? Stroke is a cerebrovascular accident; indeed, it is a brain attack and was first described at the beginning of the nineteenth century [9]. Some incidences are fatal; stroke occurs every minute world-wide [10]. Hippocrates called stroke an apoplectic seizure [11]. Yet, despite what is known about the stroke phenomenon, literature relating to cerebral neurotransmitters as the neuronal mechanics of brain/stroke, is sparse. Ischemic stroke occurs due to a loss of blood supply to a part of or all of the brain, initiating the ischemic cascade, setting into motion, a domino response of tissue factors involved in blood clots and stroke. If deprived of oxygen for more than sixty to ninety seconds and after approximately three hours, brain tissue will suffer irreversible injury possibly leading to death of a tissue, *i.e.*, infarction.

How does stroke happen? Arteriosclerosis, atherosclerotic plaques, block blood supply by narrowing the lumen of blood vessels, blood flow is decreased, blood clots form on endothelial linings of arteries and capillaries, releasing showers of small emboli through the disintegration of atherosclerotic plaques. Ultimately, pieces of partially coagulated and broken plaques *i.e.*, small emboli, float freely in the circulatory system and infarcts of apoptotic parts of the basal ganglia appear in cerebral brain tissue.

In the present paper, AIS is caused by formation of emboli derived from occlusion of the middle cerebral artery in basal ganglia of the animal brain using the nylon monofilament suture method of Zea Longa *et al.*, 1989 [12]. Clinically, embolic infarction occurs when an embolus begin its formation in the circulatory system in the heart, for example, as a consequence of atrial fibrillation. In either case, oxygen deprivation occurs, respiration becomes anaerobic, resulting in the production of oxygen free radicals, leading to cell death (apoptosis) [13–15].

Components of the apoptotic ischemic lesions are actually “alive” in an area termed “penumbra”. The penumbra is currently the focus of a goodly number of stroke investigations simply due to the fact that these salvageable living parts actually lie within the lesion or infarct. These salvageable parts of the infarct can live for twelve hours after occlusion of the middle cerebral artery [16]. The penumbra-infarct area is the neuroimaging subject of our NMI and LDF studies presented herein.

**Figure 3.**
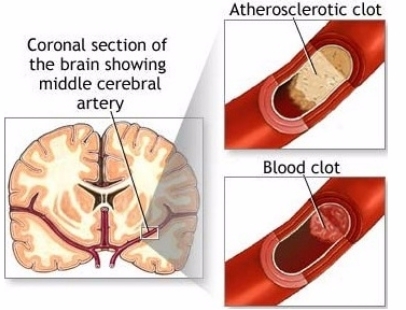
(left): schematic diagram of a coronal section of brain, the arterial composition of brain, pointing to the middle cerebral artery as it relates to basal ganglia. (right): upper and lower, schematic pictures of the endothelial lining of blood vessels forming a blood clot. Adapted from: http://stroke.about.com/od/causesofstroke/a/lacunar_strokes.htm.

An important question regarding the circulatory system is whether or not the normal hemostatic system has become pathological. Physiologic processes use the hemostatic system but infarcts are related to the pathologic component. It is the pathophysiology that reacts to blocked arteries with emboli and to dysfunctional blood vessels with aneurysms. Both hemostatic and pathologic systems need three processes to clot blood. These are *platelet*, *tissue factor* and *fibrin*. The hemostatic circulatory system uses these processes to stop excessive bleeding; the pathologic circulatory system, when in motion, produces AIS. A thrombus which surrounds the atherosclerotic plaque, taking up 75% of the space in the lumen of the artery, sets all three processes into motion. *Platelet endothelial cell adhesion molecule* deposits platelets on the wall of the lumen of the artery, a sequence of *tissue factors* generate *thrombin* that generates *fibrin* from *fibrinogen*. It is bursts of thrombin and extravasation of blood that respond to *endothelial cell wall injury* (See Figure 3 for stroke mechanism) [17].

Treatment for acute neurodegenerative diseases such as stroke and brain trauma (traumatic brain injury [TBI]) requires urgent reestablishment of blood flow to ischemic areas. Cytotoxicity has to be diminished with thrombolytic and anti-inflammatory agents. Since apoptic cells release procoagulant microparticles from the atherosclerotic plaque, infarcts must be reduced and thrombogenesis must be inhibited. Ischemia is a lack of blood flow and/or it could be a leakage of blood intracerebrally, causing a serious subarachnoid hemorrhage. Reperfusion, dissipating the thrombus in one case, clinically or opening the suture around the occluded artery in another case, *i.e.*, experimentally, will restore blood flow, but necrotic, inflammatory and cytotoxic factors released by cytokines (interleukins) occur at the same time as does reperfusion. Also, glutamate release, an excitatory neurotransmitter, triggered by calcium influx compounds the felony. Therefore, stroke is difficult to treat because reperfusion *per se* produces edema!

Consequences of stroke are extensive and varied. *i.e.*, consequences vary from inability to move one or the other side of a limb, speech deficits and inability to see in one visual field may occur [18] There are surgical means of therapy, endarterectomy is an example [19]. Sometimes the difficult decision between medicine and surgery must be made [20].

A pharmacotherapeutic means to treat AIS is enoxaparin, an antithrombotic and antiplatelet medication. We selected enoxaparin as our drug of choice for the present studies. Enoxaparin is similar to heparin. In fact, enoxaparin is low molecular weight, heparin. Like heparin, enoxaparin prevents the formation of blood clots but does so with less bleeding than does heparin [21]. Enoxaparin treatment is preferred over heparin treatment for AIS [22,23] and enoxaparin has been shown to be more effective than heparin for post-stroke thromboembolism [24]. Nonetheless, it should be noted that heparin, *per se,* is an effective medication for stroke because it is known to block leukocytes which are inflammatory molecules and thus can cause edema [25]. Moreover, other critical reasons for selecting enoxaparin for this study is its ability to reduce brain edema [26] as well as its ability to reduce cerebral infarcts, while restoring motor and cognitive impairments induced by TBI in the murine animal model [27]. Moreover, enoxaparin limits infarct size during reperfusion, the time when cytotoxic, necrotic inflammatory substances are released by atherosclerotic plaques, leading to edema [28].

Enoxaparin acts by blocking two of the twelve proteins in blood, Factor Xa and Factor II, factors which have to be active and present in order for blood to coagulate. Factor Xa inhibitors, like heparin and enoxaparin, their analogues and homologues, represent a more comprehensive approach to controlling thrombogenesis. These medications bind to Antithrombin III, a serine protease inhibitor [29]. Furthermore, enoxaparin during haemodialysis is associated with less platelet reactivity compared with heparin and is safer than heparin for patients with endstage renal disease associated with haemodialysis [30]. Enoxaparin inhibits monocyte adhesion to endothelial cells by a mechanism involving cell adhesion which leads to AIS [31]. Finally, glycoprotein GPIIb/IIIa inhibitors are direct receptor antagonists which do not allow platelets to bind to the endothelial vessel wall [32]; enoxaparin works effectively with these compounds to help stroke victims [33].

Many studies, delineating the properties of enoxaparin, were performed in the late twentieth and early twenty first centuries. In these previous studies, the excitatory neurotransmitter, glutamate, was measured as glutamate relates to calcium dependency, another critical component of atherosclerotic plaque formation [34]. The work on enoxaparin was furthered then by researchers in NYU Langone School of Medicine, who studied calcium in Purkinje fibers and suggested that enoxaparin may be a neuroprotectant [35]. French scientists studied the neuroprotective effects of enoxaparin by its synergistic response with *tissue platelet activating factor* (tPA). The therapeutic window for enoxaparin needed to halt the progression of AIS was shown to be widened suggesting a neuroprotective role for enoxaparin [36,37]. Research continued using the previous animal model to suggest a possible prophylactic role for enoxaparin wherein enoxaparin was administered prior to stroke [38,39].

In 2008, Tanne *et al.*, studied stroke medications by comparing enoxaparin with aspirin in the anti-phospholipid syndrome and found that enoxaparin remains superior to aspirin as an anti-platelet/antithrombotic [40]. Investigations into the effectiveness of tPA as a stroke medication is recently advanced by a group of German researchers who have employed *recombinant tissue plasminogen factor* (r-tPA) during the reperfusion period of the nylon suture monofilament method for middle cerebral artery occlusion [41]. These researchers performed monitoring studies on recanalization and in sonothrombolysis, contrast-enhanced ultrasound (CEUS) and found that r-tPA partially improved hemispheric perfusion and r-tPA combined with CEUS significantly reduced ischemic lesion (infarct) volume, edema and microcirculatory malfunctioning.

In the present experimental design, investigations into the efficacy of enoxaparin as a stroke medication are dramatically advanced by imaging cerebral neurotransmitters in basal ganglia using neuromolecular imaging (NMI) while at the same time monitoring cerebral blood flow with dual laser Doppler flowmetry.

## 2. Experimental

### 2.1. Conventional Voltammetry and Microvoltammetry

Significant advances in voltammetry and microvoltammetry have been made with the use of carbon fiber and carbon paste biosensors. For example, Nafion^®^ a perflourosulfonated compound, as well as ascorbic acid enzyme inhibitors are used as coatings on biosensors to separate ascorbic acid from DA. Also, a subtractogram method is used for interpreting *in vivo* recordings to separate 5-HT from DA. Therefore, brain neurotransmitter studies *in vivo* have been in progress for quite some time [42–44].

Electrochemical methods:

- Involve measurement of current as a function of applied potential, wherein
- electroactive species undergo redox reactions at a characteristic redox potential.
- Formula: O + ne^_^ ⇔ R, wherein, ne = number of electrons, O=oxidation, R= reduction.
- The current that is produced by a specific redox reaction is proportional to the concentration of neurochemicals, according to the Cottrell equation, described below.

Methods based on conventional voltammetry and microvoltammetry, as pioneered beginning in the 1970’s, have validated that the flow of charge, *i.e*., amount of current in amperes, which passes through the surface of an indicator electrode is proportional to the concentration of the electroactive species studied. The following formulas describe this relationship in terms of charge, electron transfer, current, diffusion layer, time, Faraday’s constant, size of the indicator electrode and concentration (mass) of electroactive species.

$$Q=nFVC_{oR}\;\;\;\;\;\;\;\;\;\;\;\;\;\;\;\;\;\;\;\;\;\;\;\;i=dQ/dt\;\;\;\;\;\;\;\;\;\;\;\;\;\;\;\;\;\;\;\;\;\;\;\;i=nFV\;dC_{R,t}/dt$$

where *V* is the volume of the diffusion layer on the electrode where the measurement is being made, *n* is the number of electrons transferred, *F* is the Faraday Constant, and *C_o_* denotes initial concentration. The Cottrell equation is derived from the formulas written above and demonstrates that current *i.e.*, charge and mass, *i.e.*, concentration, are proportional. The Cottrell equation is:

$$i_{t}={\mathrm{nFAC}}_{o}D_{o}{}^{1/2}/3.14^{½}{\text{t}}^{½}$$

where:

o = concentration of electroactive species oxidized.
i = current at time, t
n = number of electron transfers, eq/mol
F = Faraday’s constant, 96486 C/eq
A = electrode area, cm ^2^
C = concentration of o, mol/cm^3^
D = Diffusion coefficient of o, cm^2^/s

### 2.2. What is New about NMI? How does NMI Differ from Conventional Electrochemistry?

Firstly, NMI has developed an analyzer/detector with a reduction electronic circuit [1,2,49,51–54] wherein several neurochemicals are imaged selectively and separately each within seconds; images are recorded over months without glial formation. Secondly, NMI has already proven to meet with success in humans. Studies in epilepsy patients are published by this laboratory. NMI was used to image neurotransmitters within the temporal lobe of the human brain during intraoperative surgery for resection of neocortical tissue from patients with intractable temporal lobe epilepsy *in vivo* [45,46].

NMI provides advantages over other electrochemistry and voltammetry methods because (a) with NMI biosensors, there is no need for cumbersome head stages as are needed by conventional *in vivo* voltammetric and microvoltammetric methods [47,48] because NMI biosensors have low resistance properties [49]. (c) NMI biosensors are resistant to bacterial growth [49]. (d) Unlike carbon fiber biosensors, NMI biosensors do not form gliosis, *i.e.*, scar tissue which impedes detection of neurotransmitters and causes electrochemical signals to decay [47,48] (e) Like other carbon-paste-based biosensors, NMI biosensors respond to the lipid matrix of the brain by enhancing electron transfer kinetics; this property improves the sensitivity, selectivity and operational stability of the biosensors, allowing the detection of reliable electrochemical signals that are long-lasting [50].

**Figure 4.**
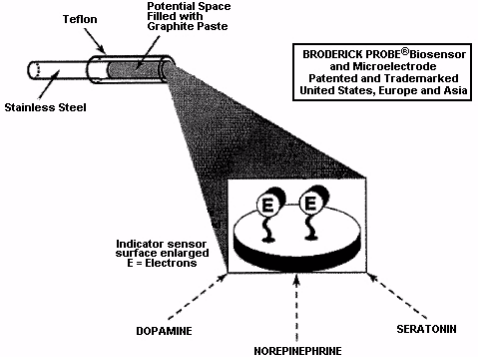
Diagram (Adapted, Broderick [49]) showing the electron transfer mechanism for NMI. Electrons are selectively transferred from neurotransmitters (NT’s) within the brain in which the biosensor is inserted. Each formulation of biosensor and NT exhibits varying hydrophilicity, hydrophobicity and polarity which will influence the cationic or anionic properties of carbon conduction which drives the electron transfer mechanism.

In this paper, results from NMI laurate biosensors are presented. Lauric acid has a hydrophobic head, hydrophilic tail, and acts as a surfactant to reduce surface tension. The surfactant, lauric acid, also acts to assist the migration of molecules to form an oriented, adsorbed film on the interfacial surface of the indicator sensor. This mechanism is a key characteristic for electron transfer kinetics exhibited by NMI biosensor subtypes [1,2,49,51–53]. Ascorbate is clearly separate from DA and 5-HT and all other neurochemicals [54]. See Figure 4 for a schematic diagram showing the electron transfer mechanism.

### 2.3. Experimental Design

In this paper, the study of the anti-platelet action of enoxaparin is advanced by imaging DStr brain neurotransmitters at the same time while the anti-platelet action of enoxaparin is monitored. The purpose is to image neurotransmitters, during control, experimental AIS, enoxaparin and reperfusion *on line*, *in vivo*, with same animal control, *i.e.*, using the animal as its own control baseline (N = 16) (Group A). Ipsilateral and Contralateral hemispheres of DStr were separately imaged and Dual Laser Doppler Flowmetry monitored Cerebral Blood Flow (Oxy Flo, Oxford Optronics, Oxford UK).

In a separate group of studies (N = 16) (Group B), quantitative histopathologies were performed in which the nylon intraluminal suture method of middle cerebral artery occlusion via midline neck incision was performed [12], this time for the purpose of delineating the areas of infarct before enoxaparin administration as well as for the purpose of delineating the diminished areas of infarct after enoxaparin and reperfusion.

After gently sacrificing the animal with pentobarbital Na (100 mg/kg., intraperitoneal (ip)) for brain dissection and for preparing brain in coronal sections, sections were placed on a laboratory glass microscope slide. Coronal sections of basal ganglia were treated with 2,3,5-triphenyltetrazolium chloride (TTC) for fifteen minutes, preserved in 10% formalin and computerized into the Image J program (National Institute of Health) for recording the percent extent of infarction before and after enoxaparin and reperfusion. Quantitative histopathology pictures are shown in the results section in the present paper.

The experimental design for NMI and LDF is seen below (studies were separately performed in each hemisphere).

- Control (non-lesioned) NMI was performed on each hemisphere of DStr at the same time as dual LDF was performed for 0.5 hour each.
- Transient focal cerebral ischemia was induced by the nylon intraluminal suture method for middle cerebral artery occlusion (MCAO) using the Zea Longa *et al.* 1989 method [12].
- NMI and LDF were continued on MCAO ipsilateral (lesioned) and contralateral (non-lesioned) hemispheres of DStr for one-half hour each.
- Enoxaparin was administered (10 mg/kg subcutaneously [sc]) during MCAO while NMI and LDF were continued on each hemisphere of DStr for one-half hour each.
- Reperfusion took place during enoxaparin treatment until the third hour and in separate studies, continuously to four and one-half hours while NMI and LDF were continued on each hemisphere.
- Animals were sacrificed on the same day.
- Infarct size (lesion) was also studied in two separate groups; one group was sacrificed at 1.5 h reperfusion time, the other at 3 h reperfusion time as shown in the present paper.

### 2.4. *In Vitro* Calibration Procedures

BRODERICK PROBE^®^ biosensors are manufactured on site. Details of the design and construction of the biosensors are published [1,2,49,51–54]. Biosensors are pre-calibrated *in vitro* in a freshly made deoxygenated physiological saline-phosphate buffer solution (0.01 M, pH = 7.4 consisting of nmol aliquots of DA, 5-HT, HVA, L-TP, ascorbic acid, somatostatin, dynorphin A (1–17) and uric acid (99% purity, Sigma-Aldrich, St. Louis, MO) [1,2,49,51–54). Moreover, calibration data from the biosensors were gleaned by substituting phosphotidylethanolamine and albumin in place of saline/phosphate buffer to mimic accurately the fat/protein composition of brain (unpublished data, in preparation for publication). Calibration curves using saline-phosphate buffer are published [56].

### 2.5. *In Vivo* Surgical Procedures

These studies are approved by the National Institutes of Health (NIH) in accordance with the Institutional Animal Care and Use Committee (IACUC) of The City College New York, The City University of New York. To begin these studies, male, Sprague Dawley, Caesarean-derived, virus-free, laboratory rats (*rattus norvegicus*) are purchased from Charles River Laboratories, Kingston, NY. When the animals arrive at our facility, they are housed in the Marshak Vivarium for about one week before surgery begins, in order to allow animals to become acclimated to their environment. Animals are fed Purina Rat Chow and water *ad libitum*. A twelve hour dark-light cycle is maintained both in the Vivarium and in Dr. Broderick’s research laboratory where studies take place, to maintain animal’s circadian rhythm. Surgery begins with an ip injection of the anesthetic pentobarbital Na (50 mg/kg in a dilute 6% solution). BRODERICK PROBE^®^ laurate biosensors are inserted stereotaxically (Kopf Stereotaxic, Tujunga, CA) within DStr (AP = +2.5; ML = +2.6; DV = −4.0) [55]: an Ag/AgCl microreference and stainless steel microauxiliary are placed in contact with dura mater. Indicator laurate biosensors are held in place with Splintline Acrylic (Lang Dental, Il.). Temperature is continuously monitored with a rectal probe and thermometer (Fisher Sci., Fadem, NJ); temperature is maintained at 37.5 ± 0.5 °C with an aquamatic K module heating pad (Amer. Hosp. Supply, Edison, NJ). Pinnal, corneal and leg flexion responses are monitored throughout surgery and supplemental doses of pentobarbital Na are administered to maintain adequate pharmacokinetic induction and depth of anesthesia. Supplemental doses of pentobarbital Na are administered as needed. The present experimental design calls for studies to be completed during anesthetic surgery. Physiologic saline (animal body weight in cc’s) is injected during this intraoperative surgery to maintain proper electrolyte and volumetric status. The total time for surgery is three to four hours.

### 2.6. Image Scanning Procedures with a Semiderivative Circuit

Potentials are applied with a CV37 detector (BAS, West Lafayette, IN) from −0.2 V to +0.9 V with respect to an Ag/AgCl (1 M NaCl) electrode, at a scan rate of 10 mV/s at time constants of 5 and 1 s tau. After charging and reduction of neurochemicals in the surrounding tissues, the indicator biosensors detect returning oxidative current from NT’s. Each scan, which includes several selectively oxidized NT’s is completed in 60 s. Nonfaradaic charging current is eliminated in the first 25 s. Relevant to these laurate studies, DA and 5-HT are detected sequentially in two waveforms with a specific signature determined by its characteristic oxidative potential (voltage) determined experimentally *in vitro*. Oxidation (half wave) potentials for these biosensors, *in vivo*, are, in the order of detection from lowest to highest; DA (0.14 V) and 5-HT (0.31 V) for the laurate biosensor. Signatures for other neurochemicals are shown in the present paper. Each specified oxidation potential is within a range of +/−0.015 V. Current is plotted as a function of potential in nano- and pico-amperes.

### 2.7. Interpretation and Analysis of Data

The current that is produced by a specific redox reaction is proportional to the concentration of each neurochemical according to the Cottrell equation explained above. Changes in concentrations of DA, 5-HT, HVA and L-TP are presented as percent changes to minimize normal between-animal variations such that each subject serves as its own control. Images were statistically analyzed using 95% Confidence Limits. P-values < 0.05 are considered to be significantly different from baseline/control values.

### 2.8. Middle Cerebral Artery Occlusion According to the Method of Zea Longa *et al.*, 1989

**Figure 5.**
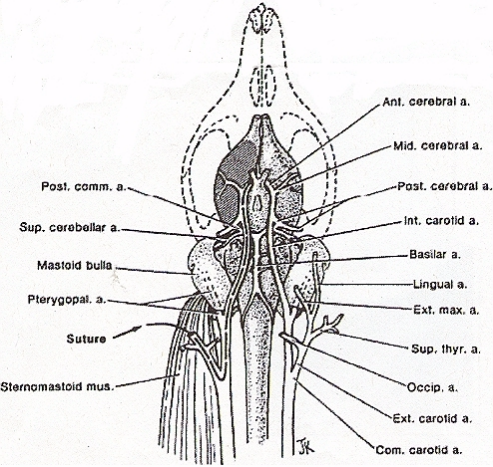
Diagram displaying the rat carotid artery (a) territories and the middle cerebral artery monofilament technique. Branches of the external carotid artery which is dissected for monofilament introduction of the suture, feed mastication and neck muscles, pharnx, tongue, salivary glands, face, external and middle ear and the meninges. Adapted from Zea Longa *et al.*, 1989 [12].

Middle cerebral artery occlusion is induced as described by Zea Longa *et al.* 1989 (See Figure 5) [12]. An operating Nikon SMZ 800 microscope (Diagnostic Instruments) with High Intensity Nova 2000 Illuminator (Morrell, Instr., LI, NY), is employed to expose the common carotid artery through a midline neck incision. The common carotid artery is then carefully dissected free from surrounding nerves and fascia, from its bifurcation to the base of the skull. The occipital artery branches of the external carotid artery are then isolated, and these branches are dissected and coagulated. The external carotid artery is dissected further distally and coagulated along the terminal lingual and auxiliary artery branches, which branches are then divided. The internal carotid artery is isolated and carefully separated from the adjunct vagus nerve and the pterygopalatine artery; the internal carotid proximal to the pterygopalatine artery is ligated close to its origin with a 6-0 silk suture. The silk suture is then tied loosely around the mobilized external carotid artery residue and a 9–10 mm in length of 7.0 nylon monofilament suture is carefully inserted through the proximal external carotid artery and gently advanced into the internal carotid artery 19–20 mm past the bifurcation of the common carotid artery and then into the Circle of Willis, effectively blocking the origin of the middle cerebral artery. Skin incision is then closed with 6-0 silk suture.

Reperfusion is achieved by withdrawal of the nylon monofilament suture from the internal carotid artery via the external carotid artery stump.

A small animal ventilator (Model 685) (Harvard Apparatus, MA) is calibrated and ready according to animals’ weight and is always on hand in case of necessity.

#### Preparation of the Monofilament Nylon Suture

The tip of the nylon monofilament suture is blunted by heating the blunted tip via a soldering apparatus. The diameter of the blunted and heated tip does not change from the original 0.2 mm diameter.

It is noteworthy that several researchers in this field of middle cerebral artery occlusion have tested different species of laboratory rodents, e.g., outcome to this occlusion procedure is limited in the spontaneously hypertensive laboratory rodent [84].

It is also noteworthy that researchers in the field of middle cerebral artery occlusion have searched for several types of modification of sutures to perform this delicate surgery, e.g., some have used silicone coating and some have used heat treated poly-L-lysine (for review, see [85]). Our method of thread manufacture is partially similar to that of Belayev *et al.* [83].

It is important to emphasize also that the penumbra has become the new focal point for treating stroke; NMI with BRODERICK PROBE^®^ biosensors are providing a valuable tool for reaching into this critical salvageable part of brain to treat stroke victims [86–88].

### 2.9. Dual Laser Doppler Flowmetry

In the present studies, dual Laser Doppler Flowmetry (LDF) measured real time erythrocyte perfusion separately in ipsilateral and contralateral hemispheres of DStr as directly related to microvascular blood flow (Oxyflo, OXFORD^™^ Ltd., UK). The stereotaxic coordinates for the placement of dual LFD optic sensors were: (AP = −1.2 mm, ML = +2.6, DV = −1.0 mm) posterior to bregma) (modified from [55]). Measurements were recorded in blood perfusion units, which are in actuality a relative and not an absolute perfusion unit scale. LDF is minimally invasive such as is NMI. However, whereas NMI operates by electron transfer, LDF operates by using a suspension of latex spheres which undergo Brownian movement; yet, this random movement is controlled with common motility standards used routinely in the public domain. With LDF, the DStr is illuminated with low power laser light from optical fiber light sensors. Laser light is scattered within the DStr and some light is scattered back to the laser light sensor. In addition, a second different optical fiber operates to collect the backscattered light from the DStr which then returns backscattered light to the perfusion monitor. Most of the light is scattered by neuronal tissue in the DStr which is immobile; some though is scattered back to mobile erythrocytes. Therefore, when the light returns to the monitor, signal processing takes the light from the *mobile* red blood cells. The blood perfusion output signal is proportional to erythrocyte perfusion. The transport of blood cells through the microvasculature is defined according to the following equation: MP = NE times VE, *i.e*.,

Microvascular perfusion = number of mobile erythrocytes multiplied by erythrocyte velocity.

Thus, BPU is equal to the product of mean blood cell perfusion and the mean blood cell velocity in the small volume of DStr, posterior to bregma, which has been illuminated by optical light sensors. The perfusion parameter is defined by information contained in the optical spectrum of light emitted from the small volume of DStr. Output to the analogue recording signal is +/−5 V.

Further analogues to LDF, NMI operates via electron transfer in a small volume of DStr to the carbon based BRODERICK PROBE^®^ biosensor according to millivolts of potential, transformed by potentiostat operational amplifiers into nanoamperes of current, which produces images of molecules comprised of NT’s. The neuroimaging parameter is defined by the specific diameter of the BRODERICK PROBE^®^ biosensor; patents covering NMI include nano-micro-millimeters in diameter [1,2]. Output to the analogue recording signal is +/−15 V [52].

## 3. Results and Discussion

### 3.1. NMI Data, Quantitative Histopathologic and Laser Doppler Flowmetry Data

**Figure 6.**
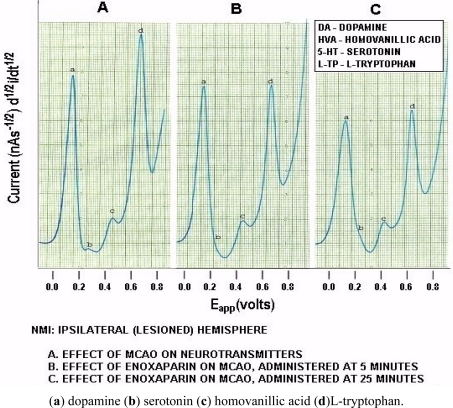
Original data of NMI after middle cerebral artery occlusion (MCAO): enoxaparin therapy, NMI from ipsilateral hemisphere.

Figure 6 below, shows actual on line, *in vivo* data which are representative of Group A, derived from NMI biotechnology and the BRODERICK PROBE^®^ laurate biosensors. The data show that enoxaparin significantly reduces the increased NT responses caused by occluding, blocking the middle cerebral artery. Scale: 10 mm = 2 na current; sensitivity setting is 5 na/volt. Data are derived from ipsilateral hemisphere. See Figure 1b for baseline/control values. Oxidation potential for HVA is 0.46 V = +/− 0.015 V; oxidation potential for L-TP is 0.68 V +/−0.015 V.

Significantly enhanced biogenic and indoleamine DA release and 5-HT release in selective electroactive, electrochemical signals and also metabolite and precursor responses respectively are the hallmarks of TBI (p < 0.05). The semiderivative circuit shows significantly enhanced DA, HVA. 5-HT and L-TP; DA response is dramatic, a ten fold increase occurs in the electroactive signal denoting dramatically enhanced DA release during middle cerebral artery occlusion. (Slide A). Enoxaparin significantly reduces the NT DA-ergic profile of TBI while still increasing 5-HT and its precursor, L-TP. Note the increase in 5-HT occurs below the x axis in slides B and C due to the electrical properties of the semiderivative circuit. DA exhibits its maximum decrease at 25 minutes after administration of enoxaparin. It is interesting too that at this time, 5-HT, HVA and L-TP begin to show downward signal processing in DStr. Literature relating to stroke and middle cerebral artery occlusion in terms of NT’s is sparse. However, one group of researchers has found a transient decrease in the DA metabolite, 3,4-dihydroxyphenylacetic acid (DOPAC) during stroke which the authors attribute to cortical degeneration [57]. The present results confirm these findings, albeit that the previous authors did not employ an *in vivo* model of assessment. At first glance, a dramatic increase in DA with a transient decrease in the metabolite DOPAC would be consistent since turnover would be slower likely due to the dramatic stroke-induced increases in the DA neurotransmitter *in vivo, per se.* Also, there are other imaging studies, such as magnetic resonance [58–60] but there is only one report that addresses DA imaging, but not release, using positron emission tomography [59]. The authors of this latter study report that the results derived, insofar as 5-HT release is concerned, are limited and ambiguous.

**Figure 7.**
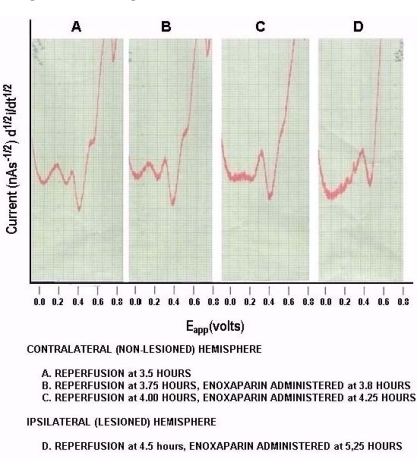
Original data of NMI after MCAO, enoxaparin and reperfusion; NMI from contralateral and ipsilateral hemispheres.

Figure 7 shows on line, *in vivo* representative data (Group A), derived from NMI biotechnology and the BRODERICK PROBE^®^ laurate biosensors. For these studies we wanted to use an experimentally different reperfusion time. Interestingly, DA and 5-HT metabolite and precursor, NT’s, show the ability to decrease TBI-associated DA systems while enhancing 5-HT systems suggesting that enoxaparin may assist in the alleviation of mood disorders which are co-morbid with stroke. DA is reduced to baseline and 5-HT is released, a ten-fold enhancement over baseline control values. In fact, atypical antipsychotic medications and 5-HT reuptake inhibitors have been shown to alleviate post-stroke depression [61,62]. Perusing the literature, one readily sees a conflict between 5-HT and stroke in that although 5-HT release can help to alleviate the depression co-morbid with stroke, there is also the possibility that administration of serotonergic compounds to stroke victims may bring about the possibility of *inducing* stroke in patients who are predisposed to cardiovascular adverse events including stroke. Another interesting relationship between serotonergic systems and stroke is the adverse gastrointestinal responses to 5-HT especially when there is an abundance of 5-HT in platelets in the gastrointestinal tract such as that seen in autistic children and in patients predisposed to stroke [63–74]. Nonetheless, the data here, given the caveat that future studies are needed, do suggest that enoxaparin may help stroke-induced depression.

**Figure 8.**
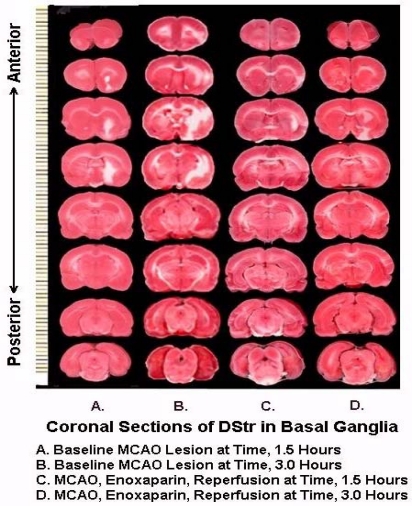
Original data of quantitative histopathologies at reperfusion, time 1.5 and 3 h.

**Figure 9.**
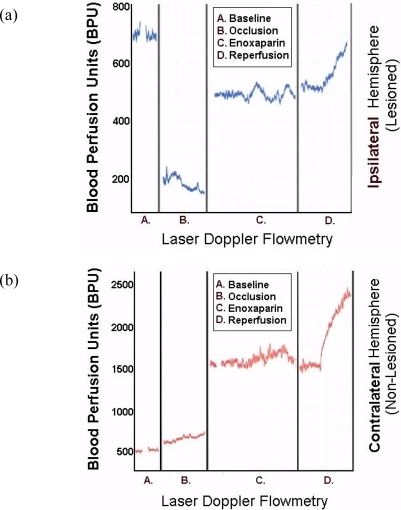
**(a)** Original data: results derived from dual LDF from ipsilateral hemisphere **(b)** Original data: results derived from dual LDF from contralateral hemisphere.

Figure 8 are representative infarcts at the 1.5 h and the 3.0 h studies of enoxaparin and reperfusion. The reperfusion is actually started while treatment with enoxaparin is ongoing. Very interestingly, enoxaparin does significantly reduce the infarcts in the DStr caused by middle cerebral artery occlusion and reperfusion produces an additional positive effect infarct reduction. This is clearly observed in the enoxaparin, reperfusion 3 h time period. The middle cerebral artery occlusion, sometimes called MCAO, was begun in 1975 by Hayakawa and Waltz wherein these researchers performed MCAO by the craniotomy method and not the midline neck incision method of Zea Longa *et al.*, 1989. The pioneers in the MCAO method are cited here [75–83]. Our results confirm the results of others; Bederson *et al.*, 1986 has published a coronal section showing the same % infarct in basal ganglia very similar to the results presented herein. The 1.5 and 3 h enoxaparin and reperfusion reduction of infarcts are clearly significant at the p < 0.05 level. Indeed, the infarcts are almost no longer visible confirming previous data that show the efficacy of enoxaparin to reduce infarct and edema as well [13–40]. Figure 9 a, below, shows the results derived from dual laser Doppler flowmetry on the ipsilateral hemisphere. Most studies have investigated only ipsilateral laser Doppler flowmetry; the previous results are confirmed [13–40].

To this author’s knowledge, the present studies are the first to present contralateral findings on line with laser Doppler flowmetry. Notably, the contralateral hemisphere shows a significant compensatory effect in response to the occlusion of the ipsilateral middle cerebral artery.

## 4. Conclusions

In summary, results show that (a) AIS produces significant enhancement of neurotransmitters DA and 5-HT, precursors and metabolites, in motor neurons of brain; an effect which occurred simultaneously with diminished cerebral blood perfusion. (b) Enoxaparin reduces DA-induced brain trauma in motor neurons while enhancing blood perfusion thereby alleviating oxygen deficiency. (c) Enoxaparin produces enhancement of 5-HT release from motor neurons within the ipsilateral hemisphere (site of lesioned hemisphere) as well as in the opposite hemisphere (contralateral) to the acute ischemic lesion. This serotonergic, possibly antidepressant effect of enoxaparin, is important because AIS is often co-morbid with clinical depression. (d) Reperfusion (removal of the arterial occlusion) significantly reduces areas of brain infarcts caused by AIS and (e) Both enoxaparin therapy and reperfusion *per se* increases cerebral blood perfusion in a compensatory manner; interestingly the response occurs in both hemispheres but more significantly within the contralateral hemisphere. Thus, this important work on behalf of stroke victims integrates the clinical aspects of neurodegenerative brain disease and presents applications of other brain technologies as well. Finally, this work shows the versatility, reproducibility and reliability of the NMI biotechnology and the BRODERICK PROBE^®^ microelectrodes/biosensors.
